# Free Vibration Analysis of DWCNTs Using CDM and Rayleigh-Schmidt Based on Nonlocal Euler-Bernoulli Beam Theory

**DOI:** 10.1155/2014/194529

**Published:** 2014-02-25

**Authors:** Maria Anna De Rosa, Maria Lippiello

**Affiliations:** ^1^School of Engineering, Viale dell'Ateneo Lucano 10, 85100 Potenza, Italy; ^2^Department of Structures for Engineering and Architecture, Via Forno Vecchio 36, 80134 Naples, Italy

## Abstract

The free vibration response of double-walled carbon nanotubes (DWCNTs) is investigated. The DWCNTs are modelled as two beams, interacting between them through the van der Waals forces, and the nonlocal Euler-Bernoulli beam theory is used. The governing equations of motion are derived using a variational approach and the free frequencies of vibrations are obtained employing two different approaches. In the first method, the two double-walled carbon nanotubes are discretized by means of the so-called “cell discretization method” (CDM) in which each nanotube is reduced to a set of rigid bars linked together by elastic cells. The resulting discrete system takes into account nonlocal effects, constraint elasticities, and the van der Waals forces. The second proposed approach, belonging to the semianalytical methods, is an optimized version of the classical Rayleigh quotient, as proposed originally by Schmidt. The resulting conditions are solved numerically. Numerical examples end the paper, in which the two approaches give lower-upper bounds to the true values, and some comparisons with existing results are offered. Comparisons of the present numerical results with those from the open literature show an excellent agreement.

## 1. Introduction

Carbon nanotubes (CNTs) constitute a prominent example of nanomaterials and nanostructures which have stimulated extensive research activities in science and engineering field. It is well known that CNTs are hollow cylindrical tubules composed of concentric graphitic shells with diameters on the scale of nanometers and, based on the number of walls, are designed as single-walled, double-walled and multiwalled nanotubes. In particular, the double-walled nanotubes can be viewed as two concentrically nested seamless grapheme cylinders bonded together by van der Waals forces.

Their discovery, since the publication of Iijima's paper [11] in 1991, has attracted much attention by many researches and several studies have shown that the carbon nanotubes possess extraordinary mechanical, physical, and electrical properties. As has been pointed out in the literature, extensive studies have been conducted on studying the mechanical properties of single-walled carbon nanotubes (SWNTs) or multiwalled carbon nanotubes (MWNTs), and several investigations have been performed by employing computational and experimental methods [12, 13]. In this context, an excellent review article on the mechanical properties of nanotubes was published by Ruoff et al. [14], in which experimental measurements as well as theoretical predictions can be found.

In the earlier studies, the investigations on carbon nanotubes have mainly focused on numerous experiments [15] although these texts, at nanoscale, are very cumbersome. In addition, two different theoretical approaches have been developed: the atomistic and the continuum mechanics. Among the methods of atomistic simulations, the classical molecular dynamic (MD) simulations is the most common method in investigating the behaviour of CNTs [16, 17]. Since this approach is restricted to small-scale systems, the continuum modelling is considered to be a more appropriate method of investigating the structural behaviour of nanotubes. In the literature, there exist a lot of studies on analysing the bending, buckling, and postbuckling problems of nanotubes using Euler-Bernoulli [18, 19] and Timoshenko [20] beam models.

In recent years, due to the remarkable importance of nanostructures for many engineering and medical devices, research interest has grown on evaluating the vibrational properties of carbon nanotubes. The literature concerning the vibrational properties in CNTs is very rich and it is devoted to the dynamic problem of single and multiwalled carbon nanotubes. In this topic, the state of the art can be found in a review work by Gibson et al. [21]. In this paper, the authors report a coherent yet concise review of as many of these publications as possible and the main themes treated are the modelling and simulation of vibrating nanotubes. Extensive studies have been conducted to investigate the vibration behaviour by means of molecular dynamic simulations: for example, Ansari et al. in [22] have analyzed the vibrations characteristics of SWCNTs and DWCNTs under various layerwise boundary conditions at different lengths. The analysis performed and the results obtained show that the natural frequency of carbon nanotubes is strongly dependent on their boundary conditions especially when tubes are shorter in length. Moreover, several researchers implemented the elastic models of beams to study the dynamic problems, such as vibration and wave propagation, of carbon nanotubes [23, 24]. Xu et al. in [9, 25], for example, studied the free vibration of DWCNTs which consist of two coaxial single-walled CNT with interacting each other by the interlayer van der Waals forces. Therefore, the inner and outer tubes are modeled as two individual elastic beams and by using the Euler-Bernoulli beam model the exact solutions for natural frequencies, at different boundary conditions, have been derived. Also Elishakoff and Pentaras [26] deal with the evaluation of fundamental natural frequencies of DWCNTs under various boundary conditions and the expressions for the natural frequencies have been derived by applying the Bubnov-Galerkin and Petrov-Galerkin methods. In the paper by Natsuki et al. [27], instead, a theoretical approach to vibration characteristic analysis of CNTs with simply supported boundary condition has been presented. Applying the Euler-Bernoulli beam theory, the authors obtained the resonant frequencies of DWCNTs with different vibrational modes and they showed that the resonant frequencies decrease with increasing length of nanotubes.

Although the classical continuum methods are efficient in performing mechanical analysis of CNTs, their applicability to identify the small-scale effects on carbon nanotubes mechanical behaviours is questionable. The importance of size effect has been pointed out in a number of studies where the size dependence of the properties of nanotubes has been investigated. For example, Sun and Zhang, in [28], discussed the scarce applicability of continuous models to nanotechnology and proposed a semicontinuum model in studying nano-materials. The authors demonstrated that the values of the Young's modulus and Poisson's ratios depend on the number of atom layers in the thickness direction. These results show that the nanostructures and nanomaterials cannot be homogenized into a continuum. At this point, the nonlocal elastic continuum models are more pertinent in predicting the structural behaviour of nanotubes because of being capable of taking in the small-scale effects. It is well known that the nonlocal elasticity theory assumes that the stress state, at a given reference point, is considered to be a function of the strain field at all points of the body. The origins of the nonlocal theory of elasticity go to pioneering works, published in early 80s, by Eringen [29]. In [30] Reddy reports a complete development of the classical and shear deformation beam theories using the nonlocal constitutive differential equations and derived the solutions for bending, buckling, and natural frequencies problems of simply supported beams.

In recent years, many researchers have applied the nonlocal elasticity concept for the bending, buckling, and vibration analysis of nanostructures. Peddieson et al. [31] have used nonlocal Euler-Bernoulli model for static analysis of nanobeams and particular attention is paid to cantilever beams which are often used as actuators in small-scale systems. Further applications of the nonlocal elasticity theory have been employed in studying the buckling problem [3, 4] and vibration problems, by applying Euler-Bernoulli beam and shell theories and Timoshenko beam theory, in CNTs [3–8, 10, 32–34].

It is worth mentioning that, in the literature, most of the attention has been focused on deriving the variational formulation of equations and boundary conditions for single- and double-walled nanotubes undergoing vibrations with nonlocal elastic continuum methods. Reddy and Pang [1] reformulated the equation of motion of the Euler-Bernoulli and Timoshenko beam theories, using the nonlocal differential constitutive relations of Eringen. Following this approach, the equations of motion are used to evaluate the static bending, vibration, and buckling responses of beams with various boundary conditions. Adali [2] proposed a continuum model for studying the mechanical behaviour of multiwalled carbon nanotubes under compressive loads; the nonlocal theory of Euler-Bernoulli beams has been employed and the results are extended to multiwalled nanotubes subjected to transverse vibrations.

In this paper the free vibration frequencies of coaxial DWCNTs are detected, using two different approaches. The first method has already been used by the authors [35] and by Raithel and Franciosi [36] for different structural problems, and it has been properly modified for the title problem. The nanotube is reduced to a set of rigid bars, linked together by elastic cells, where masses and stiffnesses are supposed to be concentrated. The resulting discrete system is simple enough to allow to take into account nonlocal effects, constraint elasticities, and van der Walls forces, and a classical eigenvalue problem is reached, which can be easily handled by Mathematica [37].

The second proposed approach belongs to the semianalytical methods, and more precisely it can be considered an optimized version of the classical Rayleigh quotient, as proposed originally by Schmidt and then employed for various eigenvalue problems [38–40]. Basically, the trial function in the Rayleigh quotient is allowed to depend on parameters, and the resulting quotient is properly optimized. The resulting conditions can be solved numerically.

Numerical examples end the paper, in which the two approaches give lower-upper bounds to the true values, and some comparisons with existing values are offered.

## 2. Theoretical Approach

**Figure 1:**
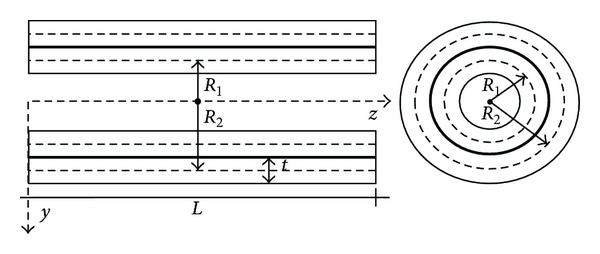
Geometry of double-walled carbon nanotubes (DWCNTs).

The beam structure, under consideration in Figure 1, is a concentric system of two nanotubes of cylindrical shape of length *L*, with Young's modulus *E* and mass density *ρ*. For each nanotube the cross-sectional area *A*_*j*_ and the moment of inertia *I*_*j*_ are defined, where the index *j* = 1,2 refers to the order of the nanotubes with the inner tube indicated by *j* = 1 and the outer tube by *j* = 2. For the DWCNTs, the main point in the analysis is the consideration of van der Waals (vdW) forces between the inner and outer tubes: the interaction pressure at any point between any two adjacent tubes depends on the difference of their deflections at that point. To take in to account the vdW forces, one defines the interaction coefficient *c*_12_ between the inner and outer nanotubes, which can be estimated approximately as [24]

(1) $$\begin{array}{c} c_{12}=\frac{320\left(2R_{1}\right)\text{erg}/\text{c}{\text{m}}^{2}}{0.16a^{2}},\;\text{with}\;\;a=1.42\times 10^{-9}\;\text{m}, \end{array}$$

where *R*_1_ is the inner radius of the wall and *a* is the carbon-carbon bond length.

As already said, the small-scale effect is taken into account by using the nonlocal theory for Euler-Bernoulli beams, so that the parameter *η* = *e*_0_ *a* is introduced, where *e*_0_ is a constant which has to be experimentally determined for each material. In turn, *a* is an internal characteristic length, as already defined.

In order to analyze the dynamic behaviour of the system under consideration, the governing equations of motion, by considering the vdW forces and the small-scale effect, have been derived using a variational approach:

(2) $$\begin{array}{c} T=\frac{1}{2}\sum_{j=1}^{2}\int_{0}^{L}\left[\rho A_{j}{\left(\frac{\partial v_{j}}{\partial t}\right)}^{2}\right]dz, \end{array}$$

(3) E1=∑j=12∫0L[12EIj(∂2vj∂z2)2−η2ρAj(∂2vj∂t2)(∂2vj∂z2)]dz+12kjRLvj′2(z=0)+12kjTLvj2(z=0)+12kjRRvj′2(z=L)+12kjTRvj2(z=L),

(4) $$\begin{array}{c} E_{2}=\frac{1}{2}\int_{0}^{L}\left[c_{12}{\left(v_{2}-v_{1}\right)}^{2}+\eta^{2}c_{12}{\left(\frac{\partial v_{2}}{\partial z}-\frac{\partial v_{1}}{\partial z}\right)}^{2}\right]dz, \end{array}$$

where *k*_*jRL*_ and *k*_*jTL*_, with *j* = 1,2, as already defined above, are rotational and translational stiffness, respectively, at *z* = 0, while, analogously, *k*_*jRR*_ and *k*_*jTR*_ are rotational and translational stiffness at *z* = *L*, respectively. In the above equations, the abscissa *z* represents the spatial coordinate while *t* is the time; in (2), (3), and (4) *T* denotes the kinetic energy, *E*_1_ is the strain energy and *E*_2_ is the potential energy due to vdW forces between the two nanotubes.

## 3. Discretization of DWCNTs by means of CDM Method

**Figure 2:**
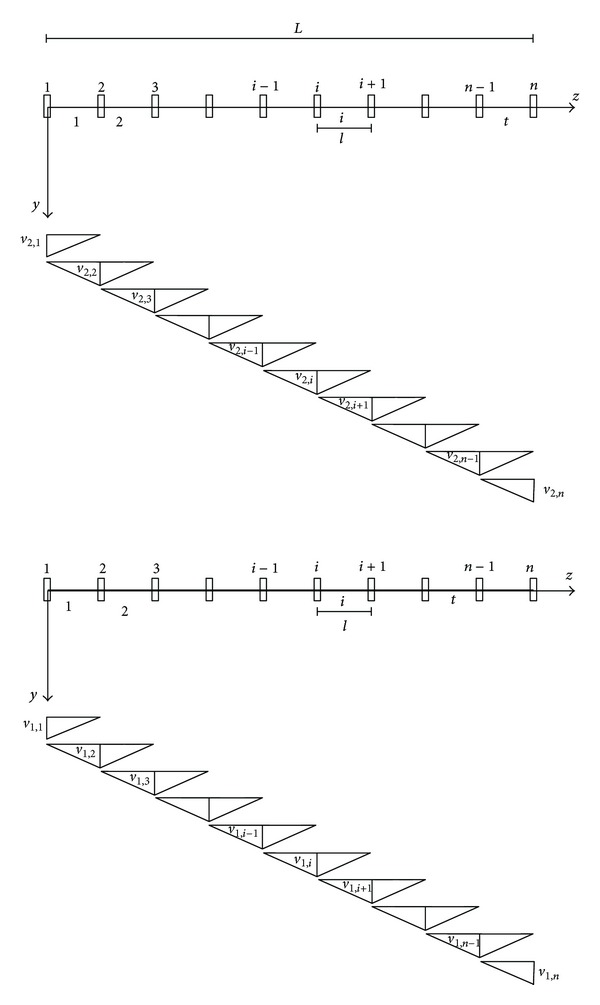
Structural system CDM.

In this section the so-called “cell discretization method” (CDM), employed to analyze the dynamic behaviour of structure under consideration, is discussed. As already said, the two nanotubes are reduced to a set of *t* rigid bars with the same length *l*, linked together by *n* = *t* + 1 elastic cells (see Figure 2). The moment of inertia *I*_*j*_ and the cross-sectional area *A*_*j*_ with *j* = 1,2 will be evaluated at the cells abscissae, obtaining the concentrated stiffness *k*_1*i*_ = *EI*_1*i*_/*l*, *k*_2*i*_ = *EI*_2*i*_/*l* and the concentrated masses *m*_1*i*_ = *ρA*_1*i*_ *l*, *m*_2*i*_ = *ρA*_2*i*_ *l* for the inner and outer tubes, respectively. Both these quantities can be organized into the so-called unassembled stiffness diagonal matrix **k**_*j*_ and the unassembled mass diagonal matrix **m**_*j*_, with dimension (*n* × *n*), *j* = 1,2, for each of two nanotubes.

In this way, the structure is reduced to a classical holonomic system, with 2*n* degrees of freedom, in particular, *n* vertical displacements *v*_1,*i*_, for inner tube, and *n* vertical displacements *v*_2,*i*_, for outer tube, at the cells abscissae will be conveniently assumed as Lagrangian coordinates and will be organized into the 2*n*-dimensional vector **v**. Moreover, for the inner and outer nanotubes the *n* − 1 rotations of the rigid bars can be calculated as a function of the Lagrangian coordinates as follows:

(5) $$\begin{array}{c} \phi_{1,i}=\frac{v_{1,i+1}-v_{1,i}}{l},\\ \phi_{2,i}=\frac{v_{2,i+1}-v_{2,i}}{l} \end{array}$$

or, in matrix form: **ϕ**_1_ = **V** **v**_1_ and **ϕ**_2_ = **V** **v**_2_ where **V** is a rectangular *transfer matrix* with *n* − 1 rows and *n* columns.

The *relative* rotations between the two faces of the elastic cells are given by

(6) $$\begin{array}{c} \psi_{j,1}=\phi_{j,1},\;\;\psi_{j,i}=\phi_{j,i}-\phi_{j,i-1},\;\;\psi_{j,n}=-\phi_{j,n-1} \end{array}$$

or in matrix form: **ψ**_1_ = Δ**ϕ**_1_ for inner rigid bar and **ψ**_2_ = Δ**ϕ**_2_ for outer rigid bar, where Δ is another rectangular *transfer matrix* with *n* rows and *n* − 1 columns.

The strain energies, *L*_*je*_ with *j* = 1,2, (the first terms of (3)), are given by

(7) $$\begin{array}{c} L_{1e}=\frac{1}{2}\sum_{i=1}^{n}k_{1,ii}\psi_{1,i}^{2},\\ L_{2e}=\frac{1}{2}\sum_{i=1}^{n}k_{2,ii}\psi_{2,i}^{2}, \end{array}$$

and they are concentrated at the cells of the inner and outer tubes, respectively.

The strain energies should be expressed as functions of the Lagrangian coordinates as follows:

(8) $$\begin{array}{c} L_{1e}=\frac{1}{2}\psi_{1}^{T}k_{1}\psi_{1}=\frac{1}{2}\phi_{1}^{T}\Delta^{T}k_{1}\Delta \phi_{1}=\frac{1}{2}v_{1}^{T}\left(V^{T}\Delta^{T}k_{1}\Delta V\right)v_{1},\\ L_{2e}=\frac{1}{2}\psi_{2}^{T}k_{2}\psi_{2}=\frac{1}{2}\phi_{2}^{T}\Delta^{T}k_{2}\Delta \phi_{2}=\frac{1}{2}v_{2}^{T}\left(V^{T}\Delta^{T}k_{2}\Delta V\right)v_{2} \end{array}$$

so that, the total strain energy can be expressed as

(9) $$\begin{array}{c} L_{e}=\frac{1}{2}v^{T}\left(\begin{array}{cc} K_{1} & 0\\ 0 & K_{2} \end{array}\right)v \end{array}$$

with **K**_1_ = (**V** ^*T*^Δ^*T*^ **k**_1_Δ**V**) and **K**_2_ = (**V** ^*T*^Δ^*T*^ **k**_2_Δ**V**). The global assembled stiffness matrix assumes the following form:

(10) $$\begin{array}{c} K=\left(\begin{array}{cc} K_{1} & 0\\ 0 & K_{2} \end{array}\right). \end{array}$$

The last term of (3), as function of the Lagrangian coordinates, assumes the following form:

(11) $$\begin{array}{c} P_{1}=\frac{1}{2}\sum_{i=1}^{n}\eta^{2}\rho A_{1,ii}{\ddot{v}}_{1,i}\psi_{1,i},\\ P_{2}=\frac{1}{2}\sum_{i=1}^{n}\eta^{2}\rho A_{2,ii}{\ddot{v}}_{2,i}\psi_{2,i} \end{array}$$

or:

(12) $$\begin{array}{c} P_{1}=\frac{1}{2}\eta^{2}{\ddot{v}}_{1}^{T}\left(m_{1}\Delta V\right)v_{1},\\ P_{2}=\frac{1}{2}\eta^{2}{\ddot{v}}_{2}^{T}\left(m_{2}\Delta V\right)v_{2}. \end{array}$$

By assembling the terms of the (12), one gets

(13) $$\begin{array}{c} P_{e}=\frac{1}{2}{\ddot{v}}^{T}\left(\begin{array}{cc} m_{1nl} & 0\\ 0 & m_{2nl} \end{array}\right)v, \end{array}$$

where **m**_1*nl*_ = (*η* ^2^ **m**_1_Δ**V**) and **m**_2*nl*_ = (*η* ^2^ **m**_2_Δ**V**). The assembled mass matrix assumes the following form

(14) $$\begin{array}{c} M_{nl}=\left(\begin{array}{cc} m_{1nl} & 0\\ 0 & m_{2nl} \end{array}\right). \end{array}$$

The kinetic energy, (2), can be expressed as the sum of the following form terms:

(15) $$\begin{array}{c} T_{1}=\frac{1}{2}{\dot{v}}_{1}^{T}m_{1}{\dot{v}}_{1},\\ T_{2}=\frac{1}{2}{\dot{v}}_{2}^{T}m_{2}{\dot{v}}_{2} \end{array}$$

or, in assembled form

(16) $$\begin{array}{c} T=\frac{1}{2}{\dot{v}}^{T}\left(\begin{array}{cc} m_{1} & 0\\ 0 & m_{2} \end{array}\right)\dot{v}. \end{array}$$

The global assembled mass matrix is given by

(17) $$\begin{array}{c} M=\left(\begin{array}{cc} m_{1} & 0\\ 0 & m_{2} \end{array}\right). \end{array}$$

The strain energy due to the vdW forces, (4), can be expressed as

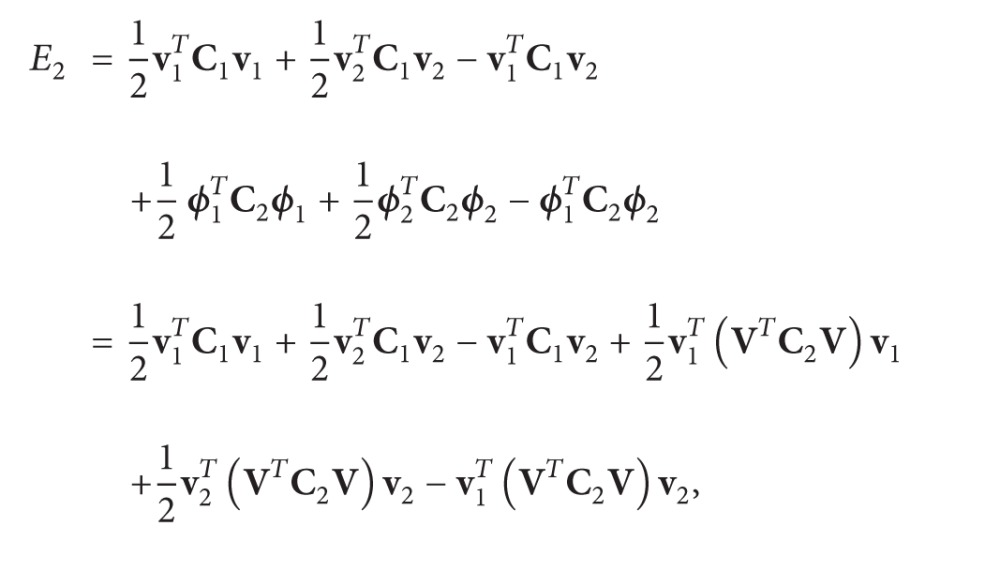
(18)
where **C**_2*a*_ = (**V** ^*T*^ **C**_2_ **V**), so that (18) becomes

(19) $$\begin{array}{c} E_{2}=\frac{1}{2}v_{1}^{T}C_{t}v_{1}+\frac{1}{2}v_{2}^{T}C_{t}v_{2}-v_{1}^{T}C_{t}v_{2} \end{array}$$

with **C**_**t**_ equal to

(20) $$\begin{array}{c} C_{t}=C_{1}+C_{2a}. \end{array}$$

The terms of matrix **C**_1_ are given by

(21) $$\begin{array}{c} C_{1,ii}=2\frac{l}{3}c_{12},\;i=3,n-3\\ C_{1,i+1,i}=C_{1,ii+1}=\frac{l}{3}c_{12},\;i=2,n-2\\ C_{1,11}=\frac{l}{6}c_{12},\;\;C_{1,22}=\frac{l}{3}c_{12},\;\;C_{1,12}=C_{1,21}=\frac{l}{12}c_{12} \end{array}$$

and the terms of the matrix **C**_2_ assume, instead, the following form:

(22) $$\begin{array}{c} C_{2,ii}=\eta^{2}c_{12}l,\;i=1,n-1. \end{array}$$

**C**_1_ and **C**_2**a**_ are two matrices with *n* rows and *n* columns and have half-bandwidths equal to 2 and they can be organized into a matrix with 2*n* rows and 2*n* columns; so that the matrix **C** takes the following form:

(23) $$\begin{array}{c} C=\left(\begin{array}{cc} C_{t} & -C_{t}\\ -C_{t} & C_{t} \end{array}\right). \end{array}$$

Finally, the strain energy terms of the flexible constraints at the ends are given by

(24) $$\begin{array}{c} L_{jTL}=\frac{1}{2}k_{jTL}v_{j1}^{2},\\ L_{jTR}=\frac{1}{2}k_{jTR}v_{jn}^{2} \end{array}$$

with *j* = 1,2, so that the assembled stiffness matrix **K** must be modified as follows:

(25) $$\begin{array}{c} K\left[1,1\right]=K\left[1,1\right]+k_{1TL},\\ K\left[n,n\right]=K\left[n,n\right]+k_{1TR},\\ K\left[n+1,n+1\right]=K\left[n+1,n+1\right]+k_{2TL},\\ K\left[2n,2n\right]=K\left[2n,2n\right]+k_{2TR}. \end{array}$$

The rotational stiffness of the constraints of each nanotube can be taken into account by summing up the corresponding flexibilities with the flexibilities of the rigid bars; for example, for the end constraints one gets

(26) $$\begin{array}{c} k_{1}\left[1,1\right]=\frac{k_{1}\left[1,1\right]k_{1RL}}{k_{1RL}+k_{1}\left[1,1\right]},\\ k_{1}\left[n,n\right]=\frac{k_{1}\left[n,n\right]k_{1RR}}{k_{1RR}+k_{1}\left[n,n\right]},\\ k_{2}\left[1,1\right]=\frac{k_{2}\left[1,1\right]k_{2RL}}{k_{2RL}+k_{2}\left[1,1\right]},\\ k_{2}\left[n,n\right]=\frac{k_{2}\left[n,n\right]k_{2RR}}{k_{2RR}+k_{2}\left[n,n\right]}. \end{array}$$

These terms will be organized in two matrices **k**_1_ and **k**_2_ furnished in (9).

Finally, the equation of motion can be written as

(27) $$\begin{array}{c} M_{t}\ddot{v}+K_{t}v=0, \end{array}$$

where **K**_**t**_ is the global assembled stiffness matrix

(28) $$\begin{array}{c} K_{t}=K+C \end{array}$$

and **M**_**t**_ the global assembled mass matrix

(29) $$\begin{array}{c} M_{t}=-M_{nl}+M. \end{array}$$

## 4. Numerical Comparisons

In order to show the potentialities of the proposed approach (CDM), several numerical examples have been performed, using a general code developed in *Mathematica* [37], and the obtained results are compared with those of available works in the literature and listed in bibliography. In the present study, the vibration analysis was carried out for DWCNTs with the same and different boundary conditions between inner and outer nanotubes, respectively. Some numerical comparisons have been performed with reference to Adali's paper [2] in which the fundamental frequencies of clamped-free double-walled nanotubes have been computed by Rayleigh-Ritz (R-R) method.

As first numerical example, the free frequencies of vibration of simply supported-simply supported DWCNTs have been calculated using as approximation function **ϕ**(*z*) = sin(*πz*/*L*) and with the vertical displacement of the inner and outer nanotubes given by

(30) $$\begin{array}{c} v_{1}\left(z\right)=a\phi \left(z\right),\\ v_{2}\left(z\right)=b\phi \left(z\right) \end{array}$$

which satisfy the boundary conditions at the simply supported ends. According to the to the Rayleigh-Ritz method, the following equation is obtained, corresponding to (45) of the paper [2]:

(31) (EI1ρA1L4ξ2ξ+c12ρA1−ω12)(EI2ρA1L4ξ2ξ+c12ρA1−A2A1ω12) −(c12ρA1)=0,

where

(32) ξ0=L−1∫0Lϕ(z)2dz,ξ1=L∫0L(dϕ(z)dz)2dz,ξ2=L3∫0L(d2ϕ(z)dz2)2dz,ξ=ξ0+η02ξ1, η0=ηL

with *η* = *e*_0_ *a*. Substituting (32) into (31), the two fundamental frequencies values are deduced. As first case, setting the interaction coefficient *c*_12_ equal to zero, one obtains the first two frequencies values, $\lambda_{i}=\sqrt{\omega_{i}^{2}(\rho A_{1}L^{4}/EI_{1})}$, for inner and outer nanotubes, respectively. In the second one, putting *c*_12_ = 0.0694 TPa (31) gives the first fundamental frequency for DWCNTs.

The simply supported-simply supported nanotubes, under consideration, have the following mechanical and geometric properties: *E* = 1.2 TPa, *ρ* = 2.3 g/cm^3^, *L* = 100 nm, *R*_1_ = 0.35 nm, *R*_2_ = 0.69 nm, and *t* = 0.34 nm, where *R*_1_ and *R*_2_ are the average radii of the inner and outer nanotubes and *t* is the thickness of the two nanotubes. In Table 1 the numerical comparisons with the results of the proposed method (CDM) and those obtained applying Rayleigh-Ritz method are reported. As it can be seen, the fundamental frequencies show that there is an excellent agreement among the results obtained by the two different numerical procedures.

This system has been already solved by Reddy and Pang [1], in the absence of van der Waals interactions, using an exact approach. On the other hand, the van der Waals forces have been taken into account in [2], where an approximate Rayleigh-Ritz method has been adopted, using a single-term trial function.

In order to check the correctness of the numerical calculations of CDM, a numerical comparison with the results given by [2] is proposed applying an optimized version of the classical Rayleigh quotient, as proposed originally by Schmidt and then employed for various eigenvalue problems in [40]. In the Rayleigh-Schmidt (R-S) method, the components of displacement and rotation as suitable analytical approximation functions are assumed, in which one or more unknown parameters are presented. For example, the two-beam displacement approximation functions can be expressed as

(33) $$\begin{array}{c} v_{1}\left(z\right)=\phi_{1}\left(z\right)+a_{2}\phi_{2}\left(z\right)+a_{3}\phi_{3}\left(z\right),\\ v_{2}\left(z\right)=\phi_{1}\left(z\right)+b_{2}\phi_{2}\left(z\right)+b_{3}\phi_{3}\left(z\right). \end{array}$$

The kinetic and strain energies and the elastic energy associated to the vdW forces can be expressed as

(34) T=12∫0L[ρA1ω2v12(z)+ρA2ω2v22(z)]dz,E1=12∫0L[EI1v2′′2(z)+EI2v2′′2(z)   −η2ρA1ω2v1v1′′(z)   −η2ρA2ω2v2v2′′(z)]dz,E2=12∫0L[c12(v2(z)−v1(z))2+η2c12(v2′(z)−v1′(z))2]dz.

After same algebra, one can write

(35) ω2=(∫0L[EI1v1′′2(z)+EI2v2′′2(z)]dz   +∫0L[c12((v2(z)−v1(z))2         +η2(v2′(z)−v1′(z))2)]dz) ×(∫0L[ρA1v12(z)+ρA2v22(z)]dz   +η2∫0L[ρA1v1v1′′(z)+ρA2v2v2′′(z)]dz)−1.

Substituting in appropriate way (33) into (34), one gets

(36) T=12ω2∫0L[ρA1(ϕ1(z)+a2ϕ2(z)+a3ϕ3(z))2       +ρA2(ϕ1(z)+b2ϕ2(z)+b3ϕ3(z))2]dz,E1=12∫0L[EI1(ϕ1′′(z)+a2ϕ2′′(z)+a3ϕ3′′(z))2      +EI2(ϕ1′′(z)+b2ϕ2′′(z)+b3ϕ3′′(z))2      −η2A1ρ(ϕ1(z)+a2ϕ2(z)+a3ϕ3(z))      ×(ϕ1′′(z)+a2ϕ2′′(z)+a3ϕ3′′(z))      −η2A2ρ(ϕ1(z)+b2ϕ2(z)+b3ϕ3(z))      ×(ϕ1′′(z)+b2ϕ2′′(z)+b3ϕ3′′(z))]dz,E2=12∫0L[c12((ϕ1(z)+b2ϕ2(z)+b3ϕ3(z))         −(ϕ1(z)+a2ϕ2(z)           +a3ϕ3(z)))2]dz  +12∫0L[η2c12((ϕ1′(z)+b2ϕ2′(z)+b3ϕ3′(z))          −(ϕ1′(z)+a2ϕ2′(z)            +a3ϕ3′(z)))2]dz.

Therefore, *ω* ^2^ depends on the unknown parameters *a*_2_, *a*_3_, *b*_2_, *b*_3_ which, in turn, can be obtained by minimizing (35). In fact, the properties of the Rayleigh quotient allow us to obtain the minimizing parameters by putting equal to zero the first derivatives of *ω* ^2^.

Let us consider a clamped-clamped double-walled nanotubes having the same geometric and mechanical properties of the Example 1. The analysis is carried out applying the Rayleigh-Ritz and Rayleigh-Schmidt methods and assuming the following approximation functions:

(37) $$\begin{array}{c} \phi_{1}\left(z\right)=1-\cos\left(\frac{2\pi z}{L}\right),\\ \phi_{2}\left(z\right)=1-\cos\left(\frac{4\pi z}{L}\right),\\ \phi_{3}\left(z\right)=1-\cos\left(\frac{6\pi z}{L}\right). \end{array}$$

In Table 3, a numerical comparison with the results given by CDM and those obtained by R-R and R-S methods is considered. As shown, the CDM results are nearer to the Rayleigh-Schmidt values than to the Rayleigh-Ritz results.

In Table 4, the fundamental frequencies of a clamped-supported double-walled nanotube are considered, for the cases *c*_12_ = 0 and *c*_12_ = 0.0694 TPa. In the first case the nondimensional frequencies *λ*_1_ and *λ*_2_ have been calculated and in the second one the value of the first frequency *λ* has been determined.

Applying Rayleigh-Ritz and Rayleigh-Schmidt methods, the approximation functions assume the following form:

(38) $$\begin{array}{c} \phi_{1}\left(z\right)=\sin\left(\frac{\pi z}{L}\right)\sin\left(\frac{\pi z}{2L}\right),\\ \phi_{2}\left(z\right)=\sin\left(\frac{2\pi z}{L}\right)\sin\left(\frac{3\pi z}{2L}\right),\\ \phi_{3}\left(z\right)=\sin\left(\frac{3\pi z}{L}\right)\sin\left(\frac{5\pi z}{2L}\right). \end{array}$$

As one can see, also this numerical example confirms that the CDM results are nearer to the Rayleigh-Schmidt values than to the Rayleigh-Ritz results.

A further numerical example, of the first free frequency of vibration and for various scaling effect parameter (*η*_0_ = 0,0.5,0.7), is illustrated in Table 5. The different values of *η*_0_ have been chosen so that possible comparisons with other references can be deduced. The results are presented for a single-walled nanotube with various boundary conditions at two ends which are of a variety of combinations, namely, simply supported-simply supported (SS-SS), clamped-simply supported (CL-SS), clamped-clamped (CL-CL), and clamped-free (CL-FR). The results given by CDM have been obtained neglecting the vdW forces. The numerical comparison has been done between the values of Tables 1–4, of the papers [4, 5] and the results given by [3]. As one can note, there is an excellent agreement between the obtained results for SS-SS, CL-SS, and CL-CL cases. In the CL-Fr single-walled nanotube case and for scaling effect parameter *η*_0_ = 0.5, the calculations provide values higher than those obtained for *η*_0_ = 0 and this is impossible. The exact values are given by the proposed method (CDM), for scaling effect parameter *η*_0_ = 0.5–0.7, and reported in the last column of Table 5.

In Tables 6 and 7, the free frequencies values for clamped-sliding end (CL-SL) and sliding end-simply supported (SL-SS) double-walled nanotubes, having the same mechanical and geometric properties of the previous examples, are reported and obtained by the CDM.

In the Table 6, the clamped-sliding end (CL-SL) double-walled nanotube case is treated. The two first nondimensional frequencies *λ*_*i*_ have been obtained for *k*_*jTL*_ = *k*_*jRL*_ = *k*_*jRR*_ = *∞* and *k*_*jTR*_ = 0 with *j* = 1,2. The case of sliding end-simply supported (SL-SS) for *k*_*jRL*_ = *k*_*jTR*_ = *∞* and *k*_*jTL*_ = *k*_*jRR*_ = 0 is reported in Table 7.

All previous numerical examples show that the nondimensional frequencies decrease as the small-scale parameter *η*_0_ increases as observed in similar studies on the free vibrations of SWCNTs [3] and DWCNTs (Tables 1, 2, 3, and 4) using the nonlocal theory.

In Table 8, a numerical comparison is illustrated between the results given by CDM and Rayleigh-Ritz methods and the results given by [6–8]. The structural system under consideration is a double-walled nanotube having the following mechanical and geometric properties: *E* = 1 TPa, *ρ* = 2.3 g/cm^3^, *L* = 14 nm, *R*_1_ = 0.35 nm, *R*_2_ = 0.7 nm, *t* = 0.35 nm, and the vdW interaction coefficient is equal to *c*_12_ = 0.0694 × 10^12^ TPa.

The numerical calculations have been performed for simply supported-simply supported, clamped-clamped, clamped-simply supported, and clamped-free double-walled nanotubes and setting the small-scale parameter equal to *η*_0_ = 0,0.1.

With reference to the paper [6], the free frequencies of double-walled carbon nanotubes (DWCNTs), $\Omega_{i}\sqrt[{4}]{\omega_{i}^{2}(\rho A_{T}L^{4}/EI_{T})}$, with *I*_*T*_ = *I*_1_ + *I*_2_ and *A*_*T*_ = *A*_1_ + *A*_2_, are calculated. The structural system is modeled following the nonlocal Euler beams theory and using the Galerkin approach. In [8], the effects of small-scale parameters on the vibrations of DWCNTs, embedded in elastic medium and based on nonlocal Timoshenko theory, are examined in detail. Finally, in [7] the fundamental free frequencies of DWCNTs have been found by means of two analytical approaches in which solving the coupled governing equations of the motion are solved.

The numerical comparisons listed in Table 8 show that there is an excellent agreement between the CDM values and those obtained in [7].

In the following numerical examples the cases of double-walled carbon nanotubes with different boundary conditions between the inner and outer tubes is considered and the obtained results are compared with the values reported in [9]. Moreover, a numerical comparison is given for the case of clamped-clamped nanotubes between the CDM and the Rayleigh-Schmidt values. The nanotubes under consideration have the following mechanical and geometric properties: *E* = 1 TPa, *ρ* = 2.3 g/cm^3^, *L* = 14 nm, *R*_1_ = 0.35 nm, *R*_2_ = 0.7 nm, and *t* = 0.34 nm, where *R*_1_ and *R*_2_ are the average radii of the inner and outer nanotubes and *t* is the thickness of the two nanotubes. The vdW interaction coefficient is equal to *c*_12_ = 71.11 GPa and the nonlocal effects are neglected. In Table 9, the first six free frequencies *ω*_*i*_ for different boundary conditions are reported. As one can see, the obtained results with CDM method are lower than those by [9].

Recently, Hemmatnezhad and Ansari [10] have furnished a finite element formulation for the free vibration analysis of embedded double-walled carbon nanotubes based on nonlocal Timoshenko beam theory. In their analysis a numerical comparison is offered with the results given by [9] and reported in Table 4 in [10] for the case of DWCNTs so constrained: the simply supported-simply supported inner tube and clamped-clamped outer tube. In Table 10 the previous numerical comparison is proposed introducing the results given by CDM and differential quadrature method (DQM), developed in [36, 41].

As can be observed the first three free frequencies *ω*_*i*_ values given by [9] overestimate the frequencies and the obtained results employing the CDM and DQM methods are lower than those by [9] while they greater than those by [10].

In Table 11, the nonlocal effects influence on the first three free frequencies of vibration are investigated, putting the small-scale parameter equal to *η*_0_ = 0.1,0.2.

The numerical calculations relative to a DWCNTs, having various lengths (14 nm, 18 nm, 24 nm, and 28 nm), are performed and the results compared with the CDM method and FEM approach given by [10]. As shown, the results given by CDM method are greater than those by [10].

In the last Tables 12 and 13 the nondimensional free frequencies values are reported for different boundary conditions among inner and outer nanotubes, respectively, and for different values of the small-scale parameter *η*_0_. The numerical calculations have been performed using the geometrical and physical data given by [2]. In particular the first nondimensional frequency value, $\lambda_{1}=\sqrt{\omega_{1}^{2}(\rho A_{1}L^{4}/EI_{1})}$, is reported. As shown, the fundamental frequencies decrease with increasing values of the small-scale parameter *η*_0_.

## 5. Conclusions

Coaxial DWCNTs are modeled as two beams interacting between then through van der Waals forces, and nonlocal Euler-Bernoulli beam theory is employed in order to calculate the free vibration frequencies of the system. Two different numerical approaches are used in order to perform numerical comparisons. In the first method, the system, under consideration, has been modeled as a set of rigid bars linked together by elastic cells, where masses and stiffnesses are supposed to be concentrated. The resulting finite degree of freedom has allowed taking into account nonlocal effects, constraint elasticities, and van der Walls forces. The second proposed approach belongs to the semianalytical methods, and more precisely it can be considered an optimized version of the classical Rayleigh quotient, as proposed originally by Schmidt and then employed for various eigenvalue problems.

Several numerical examples have been treated in detail, comparing numerical and approximate results from the literature, and the proposed approaches have furnished excellent results.

More particularly, emphasis has been given to the influence of the small-scale parameter, of the length of the nanotubes and of the various boundary conditions on the free vibration frequency behaviour.

In the author's opinion, the first method will be particularly useful—because of its intrinsic simplicity—in the future analysis of mass nanosensor and of nanotube in the presence of soil and follower forces.

## Figures and Tables

**Table 1:** Numerical comparison among R-R and CDM of simply supported DWCNTs: in columns 3 and 5 the first two dimensionless fundamental frequencies *λ*_1_ and *λ*_2_, with *c*_12_ = 0, are reported, while in column 4, the first frequency *λ*, with *c*_12_ = 0.0694 TPa, is listed.

*η*_0_	Method	*λ*_1_	*λ*	*λ*_2_
0	R-R	9.870	15.759	18.025
	CDM	9.870	15.759	18.025

0.1	R-R	9.416	15.035	17.197
	CDM	9.416	15.035	17.197

0.2	R-R	8.357	13.344	15.262
	CDM	8.357	13.344	15.262

0.3	R-R	7.182	11.468	13.117
	CDM	7.182	11.468	13.117

0.4	R-R	6.146	9.813	11.224
	CDM	6.146	9.813	11.224

0.5	R-R	5.300	8.463	9.680
	CDM	5.300	8.463	9.680

**Table 2:** Numerical comparison among [1, 2] and CDM of clamped-free DWCNTs: in columns 3 and 5 the first two dimensionless fundamental frequencies *λ*_1_ and *λ*_2_, with *c*_12_ = 0, are reported, while in column 4 the first frequency *λ*, with *c*_12_ = 0.0694 TPa, is listed.

*η*_0_	Method	*λ*_1_	*λ*	*λ*_2_
0	[2]	3.664	5.850	6.692
	[1]	3.516	—	6.422
	CDM	3.516	5.614	6.421

0.1	[2]	3.568	5.697	6.517
	[1]	3.531	—	6.449
	CDM	3.531	5.638	6.448

0.2	[2]	3.320	5.302	6.064
	[1]	3.579	—	6.537
	CDM	3.570	5.702	6.520

0.3	[2]	3.002	4.793	5.483
	[1]	3.669	—	6.700
	CDM	3.615	5.772	6.602

**Table 3:** Numerical comparison among R-R, R-S, and CDM of clamped-clamped DWCNTs: in columns 3 and 5, the first two dimensionless fundamental frequencies *λ*_1_ and *λ*_2_, with *c*_12_ = 0, are reported, while in column 4 the first frequency *λ*, with *c*_12_ = 0.0694 TPa, is listed.

*η*_0_	Method	*λ*_1_	*λ*	*λ*_2_
0	R-R	22.793	36.394	41.628
	R-S	22.410	35.783	—
	CDM	22.373	35.722	40.859

0.1	R-R	21.427	34.212	39.132
	R-S	21.137	33.750	—
	CDM	21.109	33.704	38.551

0.2	R-R	18.449	29.458	33.694
	R-S	18.303	29.225	—
	CDM	18.289	29.202	33.402

0.3	R-R	15.422	24.625	28.166
	R-S	15.359	24.525	—
	CDM	15.353	24.515	27.001

0.4	R-R	12.934	20.652	23.622
	R-S	12.907	20.609	—
	CDM	12.905	20.605	21.139

0.5	R-R	11.005	17.571	20.098
	R-S	10.992	17.552	—
	CDM	10.991	17.550	17.273

**Table 4:** Numerical comparison among R-R, R-S, and CDM of clamped-simply supported DWCNTs: in columns 3 and 5, the first two dimensionless fundamental frequencies *λ*_1_ and *λ*_2_, with *c*_12_ = 0, are reported, while in column 4, the first frequency *λ*, with *c*_12_ = 0.0694 TPa, is listed.

*η*_0_	Method	*λ*_1_	*λ*	*λ*_2_
0	R-R	15.799	25.227	28.855
	R-S	15.723	25.105	—
	CDM	15.418	24.618	28.158

0.1	R-R	14.901	23.801	27.224
	R-S	14.846	23.705	—
	CDM	14.600	23.311	26.662

0.2	R-R	12.928	20.642	23.611
	R-S	12.893	20.587	—
	CDM	12.746	20.351	23.278

0.3	R-R	10.876	17.366	19.863
	R-S	10.857	17.336	—
	CDM	10.777	17.208	19.682

0.4	R-R	9.162	14.628	16.732
	R-S	9.151	14.612	—
	CDM	9.106	14.540	16.630

0.5	R-R	7.818	12.483	13.228
	R-S	7.881	12.473	—
	CDM	7.784	12.428	14.216

**Table 5:** Numerical comparison between the results obtained with CDM and [3–5] of a SWCNT and for different values of parameter *η*_0_.

*η*_0_	Method	SS-SS	Cl-SS	Cl-Cl	Cl-FR
0	[3]	9.8696	15.4182	22.3733	3.5160
	[5]	9.8697	15.4182	22.3733	3.5160
	[4]	9.8696	15.4182	22.3733	3.5160
	CDM	9.8696	15.4180	22.3728	3.5160

0.5	[3]	5.3003	7.7837	10.9914	4.0882
	[5]	5.3001	7.7835	10.9912	4.0881
	CDM	5.3002	7.7837	10.9913	3.5874

0.7	[3]	4.0854	5.9362	8.3483	—
	[5]	4.0852	5.9362	8.3483	—
	CDM	4.0854	5.9362	8.3482	—

**Table 6:** Numerical results for clamped-sliding end DWCNTs: in columns 1 and 3, the first two dimensionless fundamental frequencies *λ*_1_ and *λ*_2_, with *c*_12_ = 0, are reported, while in column 2, the first frequency *λ*, with *c*_12_ = 0.0694 TPa, is listed.

*η*_0_	*λ*_1_	*λ*	*λ*_2_
0	5.593	8.931	10.215
0.1	5.509	8.797	10.062
0.2	5.278	8.428	9.640
0.3	4.949	7.902	9.038
0.4	4.575	7.304	8.355
0.5	4.197	6.702	7.666

**Table 7:** Numerical results for sliding end-simply supported DWCNTs: in columns 1 and 3, the first two dimensionless fundamental frequencies *λ*_1_ and *λ*_2_, with *c*_12_ = 0, are reported, while in column 2, the first frequency *λ*, with *c*_12_ = 0.0694 TPa, is listed.

*η*_0_	*λ*_1_	*λ*	*λ*_2_
0	2.467	3.940	4.506
0.1	2.438	3.892	4.452
0.2	2.354	3.759	4.299
0.3	2.232	3.564	4.077
0.4	2.090	3.336	3.816
0.5	1.941	3.099	3.545

**Table 8:** Numerical comparison between the results obtained with CDM and [6–8] of a DWCNT and for different values of parameter *η*_0_.

Boundary condition	Ω_1_	Ω_2_	Ω_3_
*Simply supported *			
*η*_0_ = 0			
[8]	3.099	—	—
[6]	3.14	6.27	9.35
[7]	3.141	6.265	9.276
C.D.M.	3.141	6.265	8.275
R-R	3.141	—	—
*η*_0_ = 0.1			
[8]	3.026	—	—
[6]	3.07	5.78	8.01
[7]	3.068	5.770	7.976
C.D.M.	3.068	5.780	8.036
R-R	3.068	—	—
*Clamped *			
*η*_0_ = 0			
[8]	4.482	—	—
[6]	4.73	7.82	10.82
[7]	4.726	7.796	10.654
C.D.M.	4.726	7.796	10.653
R-S	4.732	—	—
*η*_0_ = 0.1			
[8]	4.359	—	—
[6]	4.59	7.12	9.19
[7]	4.590	7.105	9.123
C.D.M.	4.593	7.137	9.251
R-S	4.596	—	—
*Propped *			
*η*_0_ = 0			
[8]	3.802	—	—
[6]	3.93	7.05	10.09
[7]	3.925	7.035	9.981
C.D.M.	3.925	7.035	9.981
R-S	3.965	—	—
*η*_0_ = 0.1			
[8]	3.701	—	—
[6]	3.82	6.45	8.60
[7]	3.819	6.444	8.557
C.D.M.	3.820	6.463	8.643
R-S	3.853	—	—
*Cantilever *			
*η*_0_ = 0			
[8]	1.88	4.69	7.82
[6]	1.875	4.690	7.797
C.D.M.	1.875	4.690	7.797
*η*_0_ = 0.1			
[6]	1.88	4.55	7.13
[7]	1.879	4.544	7.111
C.D.M.	1.879	4.547	7.143

**Table 9:** First free frequencies of vibrations of DWCNTs with different boundary conditions between inner and outer tubes, (for *η* = 0).

Boundary condition	Method	*ω*_1_	*ω*_2_	*ω*_3_	*ω*_4_	*ω*_5_	*ω*_6_	*ω*_7_
Fr-Fr inner	[9]	1.040	2.84	5.140	7.890	8.130	8.380	9.350
Cl-Cl outer	CDM	1.021	2.786	5.760	6.270	6.669	7.915	8.083

Fr-Fr inner	[9]	0.170	1.040	2.890	5.290	6.550	7.890	8.170
Cl-Fr outer	CDM	0.163	1.024	2.833	5.210	6.491	7.860	8.041

SS-SS inner	[9]	1.050	2.840	5.180	7.290	7.890	8.240	9.080
Cl-Cl outer	CDM	1.0219	2.789	5.104	7.212	7.914	8.243	9.010

Cl-Cl inner	[9]	1.080	2.940	5.490	7.900	8.130	8.240	—
Cl-Cl outer	CDM	1.058	2.881	5.394	7.925	8.00	8.255	9.400
	R-S	1.061	—	—	—	—	—	—

**Table 10:** Numerical comparison among [9, 10] CDM and DQM and the first three free frequencies of vibrations of DWCNTs are reported (for *η* = 0).

*L*	Mode	[10]	[9]	CDM	DQM
14	*ω*_1_	0.9097	1.05	1.0298	1.0299
	*ω*_2_	2.3601	2.84	2.7895	2.7896
	*ω*_3_	4.1481	5.18	5.1043	5.1045

18	*ω*_1_	0.5751	0.64	0.6276	0.6276
	*ω*_2_	1.4648	1.75	1.7202	1.7203
	*ω*_3_	2.6153	3.36	3.3036	3.3039

24	*ω*_1_	0.3340	0.36	0.3550	0.3549
	*ω*_2_	0.8390	1.00	0.9770	0.9767
	*ω*_3_	1.5162	1.94	1.9046	1.9046

28	*ω*_1_	0.2482	0.27	0.2614	0.2612
	*ω*_2_	0.6203	0.73	0.7200	7.1905
	*ω*_3_	1.1261	1.43	1.4074	1.4066

**Table 11:** Numerical comparison among [10] and CDM and the first three frequencies of vibrations of DWCNTs are reported (for *η*_0_ = 0.1 and *η*_0_ = 0.2).

*L*	Mode	*η*_0_ = 0.1		*η*_0_ = 0.2	
		[10]	CDM	[10]	CDM
14	*ω*_1_	0.8678	0.997	0.7103	0.866
	*ω*_2_	1.7807	2.381	1.1764	1.726
	*ω*_3_	2.7046	4.046	1.7232	2.583

18	*ω*_1_	0.5471	0.604	0.4328	0.524
	*ω*_2_	1.0966	1.459	0.7206	1.045
	*ω*_3_	1.6772	2.453	1.0936	1.563

24	*ω*_1_	0.3171	0.340	0.2446	0.295
	*ω*_2_	0.6247	0.822	0.4089	0.588
	*ω*_3_	0.9606	1.382	0.6379	0.879

28	*ω*_1_	0.2355	0.250	0.1800	0.2168
	*ω*_2_	0.4610	0.604	0.3013	0.432
	*ω*_3_	0.7103	1.016	0.4749	0.647

**Table 12:** Fundamental frequency of vibration *λ*_1_ of DWCNTs with different boundary conditions between inner and outer tubes and for *η*_0_[0.1–0.5].

*η*_0_	SS-SS inner Cl-SS outer	SS-SS inner Cl-Cl outer	SS-SS inner Cl-Fr outer	SS-SS inner Cl-Sl outer
0	24.567	35.558	24.564	35.408
0.1	23.304	33.683	23.303	33.684
0.2	20.346	29.187	20.346	29.188
0.3	17.204	24.504	17.204	24.504
0.4	14.537	20.597	14.537	20.597
0.5	12.426	17.543	12.426	17.543

**Table 13:** Fundamental frequency of vibration *λ*_1_ of DWCNTs with different boundary conditions between inner and outer tubes and for *η*_0_[0.1–0.5].

*η*_0_	Cl-Fr inner Cl-SS outer	Cl-Fr inner Cl-Cl outer	Cl-Fr inner SS-Sl outer	Cl-Fr inner Cl-Sl outer
0	24.618	35.577	8.231	8.916
0.1	23.311	33.694	8.676	8.796
0.2	20.351	29.195	8.322	8.427
0.3	17.208	24.510	7.809	7.901
0.4	14.540	20.601	7.225	7.304
0.5	12.428	17.547	6.634	6.702
